# Running the full human developmental clock in interspecies chimeras using alternative human stem cells with expanded embryonic potential

**DOI:** 10.1038/s41536-021-00135-1

**Published:** 2021-05-17

**Authors:** Justin Thomas, Ludovic Zimmerlin, Jeffrey S. Huo, Michael Considine, Leslie Cope, Elias T. Zambidis

**Affiliations:** 1 Institute for Cell Engineering, The Johns Hopkins University School of Medicine, Baltimore, MD USA; 2 Sidney Kimmel Comprehensive Cancer Center, The Johns Hopkins University School of Medicine, Baltimore, MD USA

**Keywords:** Regenerative medicine, Stem-cell biotechnology

## Abstract

Human pluripotent stem cells (hPSCs) can generate specialized cell lineages that have great potential for regenerative therapies and disease modeling. However, the developmental stage of the lineages generated from conventional hPSC cultures in vitro are embryonic in phenotype, and may not possess the cellular maturity necessary for corrective regenerative function in vivo in adult recipients. Here, we present the scientific evidence for how adult human tissues could generate human–animal interspecific chimeras to solve this problem. First, we review the phenotypes of the embryonic lineages differentiated from conventional hPSC in vitro and through organoid technologies and compare their functional relevance to the tissues generated during normal human in utero fetal and adult development. We hypothesize that the *developmental incongruence* of embryo-stage hPSC-differentiated cells transplanted into a recipient adult host niche is an important mechanism ultimately limiting their utility in cell therapies and adult disease modeling. We propose that this developmental obstacle can be overcome with optimized interspecies chimeras that permit the generation of adult-staged, patient-specific whole organs within animal hosts with human-compatible gestational time-frames. We suggest that achieving this goal may ultimately have to await the derivation of alternative, primitive totipotent-like stem cells with improved embryonic chimera capacities. We review the scientific challenges of deriving alternative human stem cell states with expanded embryonic potential, outline a path forward for conducting this emerging research with appropriate ethical and regulatory oversight, and defend the case of why current federal funding restrictions on this important category of biomedical research should be liberalized.

## Human pluripotent stem cell (hPSC) differentiation in vitro is developmentally incongruent with normal adult organogenesis

The derivation of human embryonic stem cells (hESC) from human preimplantation embryos introduced an unprecedented opportunity for directly studying early human development in vitro^1^. The subsequent derivation of patient-specific human-induced pluripotent stem cells (hiPSC)^2^ via somatic cell reprogramming bypassed the ethical concerns of deriving hESC from donated human embryos and initiated new fields of disease modeling and regenerative medicine. However, few studies have yet demonstrated long-term in vivo corrective regenerative functionality of conventional hPSC-derived cells in adult animal models or patients. Thus, the clinical utility of transplanted cells derived from in vitro hiPSC differentiation remains promising, but definitive functional restoration of diseased tissues currently remains unrealized; despite ongoing clinical trials for Parkinson’s disease, macular degeneration, retinitis pigmentosa, amyotrophic lateral sclerosis, spinal cord injury, and type I diabetes^3–5^.

The etiology of the poor in vivo function of transplanted hiPSC-derived cells currently remains elusive. However, the role of host immunity on rejection and survivability of transplanted cells derived in vitro may play at least a contributory role. Accordingly, various strategies have been employed to improve the survival of hPSC derivatives in vivo (e.g., the use of hypo-immunogenic hPSC lines and biomaterial delivery of tissue transplants), but with only partial amelioration of the problem^6–8^. The ineffectiveness of conventional hPSC-derived cells transplanted into adults is likely multivariate and includes at a minimum, cell culture-associated enzymatic mechanical disruptions, or epigenetic artifacts that may destabilize survival or genomic integrity in vivo. For example, the prolonged culture of conventional, primed hPSC lines promotes abnormal karyotypes and aberrant X-inactivation^9^. In addition, acquisition of donor cell epigenetic memory, ineffective/incomplete reprogramming, and leaky lineage-primed gene expression impact and diminish the potency and quality of differentiation from conventional hiPSC lines^10^.

However, correction of these cell culture-associated caveats may still not improve the inherent problem of poor function of embryonic cells transplanted into adult recipients. There is abundant evidence of a “developmental incongruence” of embryonic cells for functional engraftment within developmentally incompatible adult niches. For example, efficient in vivo engraftment and chimera progression were possible only when injected embryonic stem cells closely matched the embryonic developmental stage of the recipient host embryo^11^. Similarly, although hPSC-derived cardiomyocytes closely phenotype fetal tissue cardiomyocytes, both were incompatible for transplantation into adult hosts due to developmental differences in contractile force, metabolic regulation, microtubule organization, and electrophysiology^12^.

Interestingly, the allure of current hiPSC-derived cell replacement strategies resembles the historic efforts of fetal tissue transplantation; which were ultimately abandoned due to lack of clinical efficacy, as well as ethical dilemmas associated with procuring fetal tissues^13^. Like fetal tissues, hiPSC-derived embryonic progenitors have the capacity for extensive regenerative plasticity, proliferation, and potentially low allogeneic immunogenicity. However, transplanted hiPSC-derived cells, like fetal tissues, may similarly suffer poor engraftment and functionality due to an embryonic developmental incongruence with adult host recipients.

Herein, we advance the argument that *a potentially critical variable limiting the functionality and ultimate utility of* in vitro*-differentiated hPSC-derived cells is their embryonic-staged developmental incompatibility with an adult recipient host*. We hypothesize that immutable, species-specific developmental clocks requisitely progress to adult stages *only* within appropriate *adult* niches, following stage-specific organogenesis cues^14–19^. We suggest that this obstacle may ultimately restrict the capacity of conventional hPSC for producing clinically relevant, adult-staged tissues in vitro, including via organoid technologies. We also posit that developmental incongruence between embryonic disease-affected hiPSC-derived cell lineages and their adult diseased counterparts may also ultimately limit their utility for modeling adult pathologies (e.g., age-related neurodegenerative disorders)^20^.

We define a framework for understanding this barrier to clinical application of hPSC-derived tissue via the “*developmental incongruence*” hypothesis. We suggest that the solution to this major caveat in the fields of regenerative medicine and human disease modeling is the optimization of human–animal interspecies chimera technology, which could potentially generate fully developed, mature, patient-specific whole organs within adult host animals. Finally, we propose that more primitive, totipotent-like human stem cells will first need to be derived to achieve the embryonic chimera efficiency necessary to unlock the full developmental potential of patient-specific stem cells.

## In vitro PSC differentiation faithfully recapitulates the ordered, sequential stages of embryonic gastrulation

**Fig. 1:**
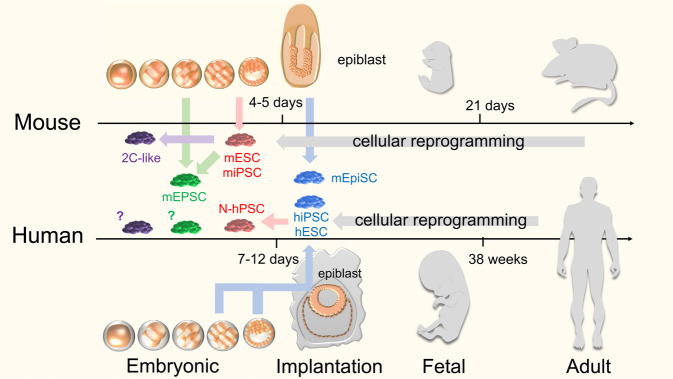
Comparative incongruence of murine and human embryonic and developmental stages. *Top:* Murine ESC (mESC; red) are derived from pre-implantation morula- or blastocyst-staged murine embryos, and capture a naïve epiblast-like pluripotent state. mESCs can differentiate into all three germ-layers via in vitro directed differentiation, in vivo teratomas, and in vivo morula/blastocyst chimera assays. The murine pluripotent state can also be obtained through epigenetic reprogramming of somatic cells with ectopic expression of Yamanaka factors. Murine extended potential stem cells (mEPSCs; green) possess both embryonic and extra-embryonic differentiation potency and can be derived via chemical reprogramming of cleavage-stage murine blastomere cells of mESCs. Stem cells resembling the molecular phenotype of 2-cell (2C) cleavage-staged blastomeres (purple) putatively possessing totipotent-like molecular characteristics can be isolated from mESC cell cultures^122^, or through the inductive expression of key totipotent factors (e.g., *Dux* and *NELF-A*). *Bottom*: Although conventional hESC (blue) are similarly derived from the pre-implantation stages of IVF-derived morula or blastocyst-staged human embryos, they are phenotypically similar in culture with post-implantation primed murine epiblast stem cells (mEpiSC; blue). A primed, mEpiSC-like state of pluripotency is also attained through the epigenetic reprogramming of human somatic cells by Yamanaka factors into hiPSC. Multiple methods for reverting conventional hPSC to naïve human pluripotent stem cells (N-hPSC; red) with mESC-like pluripotency have been reported employing various small molecule inhibitor cocktails or via transgene expression of key factors^10^. Self-renewing human totipotent stem cells (purple) with phenotypic equivalence to four to eight-cell or cleavage stage human blastomeres have not yet been described.

Although our knowledge of the requisite signals for early embryonic lineage specification has grown in the past 40 years, a full and accurate understanding of human fetal organogenesis remains elusive. The parallel uses of murine pluripotent stem cell (PSC) in vitro modeling, paired with in vivo murine embryo lineage tracing have revealed the main developmental stages and key signals that determine conserved early lineage fate commitment in both rodents and humans^21^ (Fig. 1). Doetschman et al.^22^ reported the first system for in vitro germ layer differentiation of murine embryonic stem cells (mESC)^22^. Aggregated mESC suspension cultures produced multi-lineage cystic structures termed embryoid bodies (EBs) that modeled post-implantation gastrulation events. Prolonged EB culture generated endodermal proteins (e.g., alpha-fetoprotein) and mesoderm-derived myocardium and erythrocytes.

Similarly, in vitro, human EB differentiation from hPSC was shown to replicate the extrinsic signals that were required in vivo to guide sequential bifurcation stage fate decisions during human gastrulation^23^. Biochemical agonizts or antagonists (e.g., Activin/Nodal/TGF-β, BMP, WNT, FGF, and NOTCH signaling), stromal cell co-culture, and use of extracellular matrix (ECM) substrates at various developmental junctures were rationally employed to mimic the signaling gradients required for segmentation of the gastrulating human embryo toward anterior, posterior, dorsal, and ventral planes; consequentially leading to germ layer lineage commitment (i.e., mesoderm, endoderm, and ectoderm). For example, primitive streak (PS) formation was recapitulated faithfully in vitro through simultaneous use of WNT3A and ActivinA/Nodal signaling. hPSCs differentiated into Brachyury (T)- and FOXA2-expressing populations (i.e., T^pos^/FOXA2^high^, T^pos^/FOXA2^medium^, and T^pos^/FOXA2^low^), representing the anterior, mid, and posterior segments of the PS, respectively. Cardiac mesoderm arose from T^pos^/FOXA22^medium^ PS populations through attenuated WNT and FGF signaling, while combined WNT and BMP4 signaling skewed differentiation toward a posterior hematovascular mesoderm fate. Alternatively, Nodal signaling of PS populations skewed hPSCs toward the anterior endoderm.

## In vitro differentiation of PSC to embryonic hematopoietic lineages is efficient, but fails to generate long-term engrafting, adult-stage hematopoietic stem cells (HSC)

**Fig. 2:**
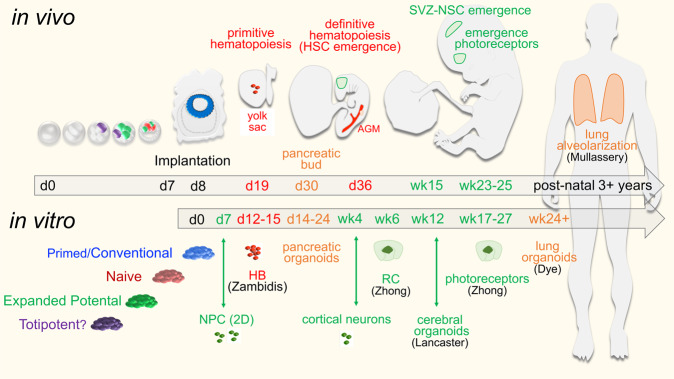
Correlation of normal human developmental kinetics and its developmental congruence with in vitro hPSC directed differentiation. Mesoderm. The first wave of human embryonic hematopoiesis begins in the yolk-sac at ~day 19 while self-renewing HSC emerges during the sixth week of gestation in the aorta-gonad-mesonephros (AGM)^123^. Embryonic hematopoietic waves arise from yolk-sac-like hemangioblast (HB) progenitors after 12 days of in vitro hematopoietic differentiation of hPSC^29,30^. Yolk sac-like hemogenic endothelium (HE) can similarly be derived via hPSC in vitro differentiation protocols after 15 days of hematopoietic differentiation^29,32^. Long-term engrafting HSCs similar to those arising from human fetal AGM have not yet been generated via in vitro hPSC differentiation. Endoderm. Pancreatic organoids can be generated in vitro from hPSC with early beta-islet and acinar progenitor cells between 14 and 24 days, and correlate with the emergence of the fetal pancreatic bud at ~day 30^51,52^. Lung alveolar structures generated in vitro from hPSC after 24 weeks of continuous culture resemble transcriptional phenotypes of fetal lung tissues^124,125^. Ectoderm retinal cup (RC) and photoreceptor differentiation can be recapitulated in vitro via hPSC-based retinal organoid protocols at ~6 and 17 weeks, respectively, and parallel in vivo retinal developmental events^63,126^. Post-natal adult neural stem cells emerge in utero in the sub-ventricular zone (SVZ) during week 15 of gestation, and their development can be recapitulated within cerebral organoids, albeit with limited progression to adult neural maturation^58,127^.

Much of our understanding of hematopoiesis originated from lineage tracing experiments in murine embryos. Murine hematopoiesis arises in two waves: the first at ~E7, in the murine yolk sac, called (embryonic) primitive hematopoiesis; the second at ~E8.5, in the aorta-gonad-mesonephros (AGM), termed (adult) definitive hematopoiesis^24^ (Fig. 2). Functional hematopoietic stem cells (HSCs) can only be isolated from the AGM-derived adult-type definitive hematopoietic wave at ~E10.5 of murine development. These HSC are functionally validated by their capacity for long-term rescue and repopulation of the entire hematopoietic system of lethally-irradiated adult mice, in both primary and secondary HSC transplantation assays. A similar pattern of hematopoiesis exists within human embryonic and fetal development but on a more extended timeclock. Primitive erythro-myeloid hematopoiesis first arises during days 16–17 of human development within the yolk sac, while HSCs arise at low frequency after ~3–5 weeks of human gestation from the human fetal AGM^25^ (Fig. 2). However, the full developmental sequences of human hematopoiesis remain obscure due to the inaccessibility of human fetal tissues.

One important goal of regenerative medicine is to derive unlimited supplies of clinically useful, mature, AGM-like adult, long-term repopulating HSC from PSCs. Toward addressing this challenge, in vitro differentiation of mESCs to the hematopoietic fate confirmed the hypothesis that primitive blood cells and their surrounding endothelium in the yolk sac were derived from a common hemato-endothelial ancestor termed the hemangioblast^26–28^. Hematopoietic differentiation from hPSCs similarly produced human hemangioblasts that gave rise to yolk sac-like embryonic waves of hematopoiesis and expressed key transcriptional factors (e.g., *SCL*, *GATA1*, *GATA2*, and *CDX4*) and markers of in vivo embryonic hematopoieisis^29^. A second in vitro embryonic wave was characterized by expression of the cell surface markers CD34 and CD45, and correlated with a more mature, but still yolk sac-like “definitive” embryonic hematopoietic lineage that did not have the characteristics of bona fide adult definitive, AGM-like erythropoiesis (e.g., high expression of beta-globin hemoglobin chains)^29^. By tracing the expression levels of angiotensin-converting enzyme (ACE) during EB differentiation, Zambidis et al.^30^ marked the key hemangioblastic transition of the PS of hematopoiesis toward a hemato-endothelial phase beginning at Day 12 of differentiation. Importantly, it was noted that there was temporal alignment between the days of culture required to transition hematopoietic progenitors in vitro that aligned with the same post-implantation events in the human PS. Human hematopoietic differentiation both in vivo and in vitro followed a temporal sequence beginning with a primitive wave of progenitors expressing embryonic hemoglobins, followed by a wave of yolk sac-like myeloid-lymphoid development. In utero, these temporal waves progressed within defined yolk sac, and not adult-like definitive (e.g., AGM, dorsal aorta, or fetal) hematopoietic niches (Fig. 2). This is an important conceptual distinction because yolk sac progenitors do not possess HSC self-renewal, and unlike AGM-derived HSC that expand in the fetal liver, they are unable to engraft efficiently to reconstitute multi-lineage lymphoid-hematopoiesis in an adult environment.

To bypass in vitro differentiation barriers that restrict hPSC to embryonic hematopoiesis, and to test the hypothesis that a developing hemato-vascular microenvironment might provide the niche signals required for maturing hPSC-derived progenitors to adult-type hematopoiesis, Park et al.^31^ injected hESC-derived CD34^+^ hematopoietic progenitors into the vasculature of chicken embryos and allowed them to engraft for up to 14 days. Although engraftment of human progenitors within the hematopoietic organs of the chicken (e.g., bursa Fabricius and thymus) was observed, these progenitors showed limited maturation into adult erythro-myeloid or lymphoid lineages. For example, erythrocytes derived from engrafted hPSC-derived CD34+ progenitors did not produce significant expressions of adult β-hemoglobin chains.

Additional studies similarly tested the hypothesis that missing adult extracellular niche signals could mature embryonic hematopoiesis derived from hPSCs into adult stage HSC. For example, differentiation was performed on the substrate Tenascin C, an ECM protein secreted by mesenchymal stroma in the AGM and bone marrow. Although the increased frequency of erythro-myeloid colony-forming cell assays and T-lymphoid commitment was demonstrated from Tenascin C-cultured hPSC-derived hemato-endothelial progenitors, AGM-like adult-stage HSC could not be generated^32^. Other studies similarly demonstrated that hPSC-derived primitive hematopoietic progenitors could be expanded or advanced to more mature embryonic stages via stromal culture or even in vivo teratoma hematopoietic differentiation that generated progenitors with multi-lineage adult engraftment capacity^33–35^. However, these approaches were collectively unable to generate adult-stage long-term engrafting HSC with convincing evidence of self-renewal following their transplantation into developmentally mature hematopoietic micro-environmental niches^36,37^.

As an alternative to in vitro niche signaling manipulation, deterministic transcription factor genetic reprogramming was further employed to reprogram embryonic PSC-derived stem-progenitors capable of rescuing lethally irradiated mice. For example, mESCs expressing an ectopic *homeobox B4* (*HoxB4)* transgene directed primitive hematopoietic progenitors toward a definitive HSC phenotype that reconstituted irradiated adult mice with predominantly adult myeloid progeny and adult-like erythrocytes expressing β-globin chains^38^. However, ectopic HOXB4 expression, as well as its downstream target CXCR4, did not impart an adult-type engrafting HSC phenotype upon hPSC-derived hematopoietic progenitors^39,40^. Interestingly, ectopic expression of the leukemic fusion protein MLL-AF4 was sufficient to generate long-term engraftable HSC, but not surprisingly also conferred malignant potential upon them^41^.

In more sophisticated factor reprogramming studies, Sugimura et al.^42^ discovered a combination of seven genes (i.e., *ERG*, *HOXA5*, *HOXA9*, *HOXA10*, *LCOR*, *RUNX1*, and *SPI1*) that when induced within hESC-derived embryonic stage hemogenic endothelial progenitors generated HSC that were capable of both primary and secondary BM reconstitution of irradiated immune-compromised mice^42^. In addition, factor-programmed progenitors produced adult enucleated erythroblasts with an expression of adult β-hemoglobin comparable to that generated by cord blood HSCs (CB-HSCs). Interestingly, although these and other^43^ progenitors derived by forwarding transgenic programming behaved functionally as HSCs, their transcriptional signatures did not align with that of bona fide CB-HSCs, thus possibly signifying an incompletely reprogrammed, intermediate embryonic state.

These and many other studies not reviewed here demonstrated that although maturation of embryonic hematopoietic progenitors to a more mature embryonic state was feasible^44^, full development into adult AGM-like HSC by exposure to in vitro signals alone ultimately failed. The generation of hPSC-derived adult-staged HSC with the ability to produce enucleated erythrocytes expressing alpha/beta hemoglobin chains, B and T lymphocytes with mature VDJ rearrangements, and the capacity for long-term reconstitution in irradiated adult recipients in primary and secondary transplantation assays currently remains elusive via in vitro differentiation paradigms. Thus, until the challenge of generating adult-stage HSCs with long-term repopulating functionalities is overcome, the use of hPSC for hematological therapeutics will continue to remain limited.

Nonetheless, it is important to emphasize that embryonic hiPSC-derived blood progenitors could still offer unique and significant therapeutic potential in adult recipients. For example, the proclivity of hiPSCs for embryonic hematopoiesis may allow the mass production of embryonic myeloid progenitors from hPSC capable of terminal differentiation into primitive microglia and alveolar macrophages with long-term engraftment in mice^45–47^. These hiPSC-derived resident primitive macrophages were capable of alveolar surfactant clearance in the lungs of mouse models of hereditary pulmonary alveolar proteinosis^48^. Additionally, platelets from embryonic hiPSC-derived progenitors may possess functionally similar to adult platelets, but with potentially reduced immunogenicity^49,50^.

## Pancreatic differentiation to adult developmental stages remains limited in vitro

Endoderm arises from T^pos^/Foxa2^high^ populations in the anterior portion of the PS and gives rise to lungs, liver, and pancreas^21^. PSC-derived in vitro pancreatic differentiation via EB methods can generate islet-like structures expressing endocrine cell (EC) hormones insulin, glucagon, and somatostatin (Fig. 2). However, these structures failed to bring streptozotocin (STZ)-diabetic mice back to euglycemia. Improved pancreatic differentiation protocols subsequently promoted developmental maturation and survival of ECs after transplantation, with improved glucose-stimulated insulin secretion (GSIS)^51^. For example, combined Nodal, Activin A, and WNT signaling efficiently induced definitive endoderm differentiation. Subsequent replacement of Activin A for FGF signaling potentiated primitive gut tube differentiation; SHH and BMP inhibitory signals instead directed endoderm to differentiate into liver and lung tissues. Further improvements utilized hormones, growth factors, and chemical signaling agonist/antagonist to induce and expand EC to pancreatic progenitors^51,52^. Despite these cell culture optimizations, PSC-derived β-islet-like cells retained developmental immaturity, did not match islet cells that develop naturally in vivo, displayed limited GSIS in vitro, showed divergent transcriptomes to adult counterparts, and had low survivability following transplantation. Nonetheless, in vitro-derived β-islet-like cells still displayed promising amelioration of hyperglycemia in mice at ~4 months post-transplantation. Several promising clinical trials focusing on the safety of transplanting hPSC-derived pancreatic progenitors into Type 1 diabetic patients are currently ongoing but with as yet unreported long-term reversal of diabetes^51^.

## Ectodermal lineage commitment from PSC-differentiated cultures primarily recapitulates embryonic neurulation

Unlike the developmental trajectories of mesoderm and endoderm, in vitro neuroepithelial (NE) commitment proceeds spontaneously from PSC without additive morphogens, within a timeline congruent to that of spontaneous neural tube development in utero (Fig. 2). PSCs pattern into an anterior cell population that develops into the forebrain, and increased WNT gradient signaling subsequently commits NE to either posterior midbrain or hindbrain lineages, respectively. This in vitro patterning occurs within 3 weeks, with similar timing as It does in utero. Intrinsic morphogenic signaling of NE differentiation can be mimicked by dual TGF-β inhibition (SB431542) and BMP antagonism (Noggin). Further SHH signaling can terminally differentiate midbrain-neural progenitors into dopaminergic neurons^53–55^. These in vitro protocols required an ~5-week time period, and recapitulated innate temporal kinetics in utero. However, terminally differentiated neurons required several months to fully mature, form functioning neural networks, and produce rhythmic action potentials. This necessity of prolonged in vitro culture for producing functional neural networks is ultimately a limiting caveat for disease modeling and drug discovery. For example, although neurodegenerative diseases such as Parkinson’s and Alzheimer’s target specific subtypes of neurons, the pathophysiology occurs in the milieu of diverse networks of tangentially related cell types that develop primarily in (elderly) adults.

## Three-dimensional (3D) organoid differentiation systems significantly advance but do not resolve the obstacles of the limited development of hPSC toward mature organogenesis

The observation that hiPSC-derived teratomas gave rise to engraftable adult-like hematopoietic progenitors suggested that continued exposure to a multi-lineage teratoma milieu could bypass the limitations of two-dimensional (2D) in vitro neural differentiation^54,55^. To better understand the nature of how species-specific developmental clocks within PSC may operate ex vivo, Barry et al.^56^ performed parallel neural differentiation of hESC and mouse epiblast stem cells (mEpiSC) in vitro and in vivo with 3D teratoma assays. Notably, the use of mEpiSCs minimized confounding variables that may arise from the differing pluripotency of lineage-primed conventional hESC and mESC. Human and murine neural progenitor samples were periodically evaluated from differentiated cultures. Time-lapsed expression analysis (RNA-seq) revealed thousands of lineage-associated neural progenitor genes that were expressed with more rapid kinetics in mice than in human differentiation cultures. To control for the possibility that in vitro conditions technically influenced the transcriptional primacy of differentiated cells, neural progenitors derived from mature teratomas from both mEpiSC and hESC were characterized in parallel. Murine neural progenitors from teratomas again expressed neural-specific genes with faster kinetics than their human counterparts. Importantly, the differentiation kinetics of teratoma-derived neural progenitors not only mimicked those of in vitro-derived neural progenitors exposed to mature tissue and growth factors, but also the kinetics of normal in utero neural tube formation (Fig. 2). These studies verified parallel kinetics between in vitro PSC differentiation, teratoma formation, and natural fetal development; similar to the observations made with ex vivo human embryonic hematopoiesis^29^.

Further efforts to address the limited maturation to inter-connected adult–cellular subtypes in 2D hPSC differentiation cultures led to the development of sophisticated stem cell-based 3D organoid culture systems^57^. For example, Lancaster et al.^58^ derived a morphogen and mitogen-free 3D culture system that differentiated hPSCs into aggregated “cerebral organoids” that spatiotemporally recapitulated the regional development of the brain^58^. Neural progenitors organized into ventricular zones, subventricular zones, and cortical plates that paralleled in utero development of the fetal brain. Further signaling modifications produced regional cerebral organoids mimicking the organization and cross-talk of the ventral fetal forebrain. Interestingly, neural projections from cerebral organoids could innervate murine perispinal cord muscle circuitry to produce muscle contraction^59^.

Although these ex vivo organoid systems opened new avenues for exploring the organized physiological potential of hPSC-derived tissues, long-term growth and maturation were limited and susceptible to batch-batch experimental variation. For example, a comparison of glial stem cells from primary human cortex and those generated from mature cerebral organoids revealed a developmental incongruence with organoids possessing less diverse and less differentiated neuronal populations^60^. In addition, cerebral organoids could not reproduce organized human laminated neuronal regions. Instead, in vitro-derived organoids possessed random juxtaposition of distinct neuroanatomic areas^61^; albeit limited maturation was somewhat improved by transplanting organoids in vivo into mouse brain^60^. Other studies comparing gene expression signatures of iPSC-derived human or murine retinal organoids to fetal/adult retinal tissues revealed similar temporal developmental incongruences and deficiencies with normal adult retinal photoreceptors^62,63^.

Furthermore, although early neurogenesis in vitro and in vivo both occurred in the absence of vascularization, as these tissues matured beyond the capacity of passive oxygen diffusion, blood vessels are necessary to provide the oxygen and nutrients that support viable proliferation. This absence of nutrient flow limited the growth of cerebral organoids to less than 5 mm, and subsequently led to necrosis at the structures’ cores. This limitation was partially ameliorated with approaches employing micro-chip implant co-cultures of organoids with vascular endothelial cells, in vivo transplantation, and slice cultures prolong viability^64^. Thus, the generation of complex, connected neural circuits within cerebral organoids currently remains elusive and limited primarily by lack of adult-type neuronal myelination^65^. In summary, although organoid technologies represent a promising and improved alternative to 2D directed hPSC differentiation, these systems currently only partially recapitulate normal in vivo human organogenesis.

## Do innate cell-autonomous developmental clocks of hPSC ultimately impede the in vitro generation of adult-staged cells for regenerative medicine?

Current in vitro differentiation paradigms focuses on replicating extrinsic signaling of fetal development to guide progenitors through a “natural” developmental timeline. Such a model primarily views differentiation as a spatial occurrence that is dependent on environmental niche cues (i.e., extrinsic factors). The studies reviewed above suggest that hiPSC-derived embryonic progenitors maintain stochastic epigenomic plasticity dictated by developmental timing that is only partially malleable by artificial micro-environments.

Alternatively, it is possible that the shortcomings of in vitro hPSC differentiation may primarily be attributed to the context in which we conceptualize in vivo developmental biology. For example, cell states have a nuanced program of differentiation that challenges the notion of fixed, binary lineage-specification decisions that can be coaxed by in vitro manipulations. Multiple intermediary states may exist between progenitors and their terminally differentiated outcomes^66^. Completion of the necessary cell cycles required to achieve terminal differentiation may be at the core of progression to adult differentiation. However, collectively, these and the other studies comparing species-specific developmental tempos support an alternate hypothesis: immutable species-specific developmental clocks are innately programmed into human and murine PSC in a cell-autonomous manner^14,15,67^.

Differentiation could therefore be modeled with reliance on not only cell-extrinsic factors (e.g., extracellular matrices, cell morphogens, small molecule inhibitors, mesenchymal niche cells, and the length of time in a culture of stem cells ex vivo, but also by immutable, cell-intrinsic factors (e.g., developmental clocks). The mechanisms that drive such autonomous developmental clocks in stem cells are obscure. However, progression to adult organogenesis may ultimately be impossible in vitro if species- and stage-specific niche signals are available only over long periods of time (e.g., months–years) in the adult, and are not malleable by ex vivo acceleration with ectopic biochemical/genetic signals. Indeed, Rayon et al.^14^ suggested that innate developmental tempo may be regulated by factors that cannot be directly manipulated; such as species-specific protein stability and cell cycle duration. Thus, innate developmental clocks programmed into PSC may be thought of per se as the most critical variable of differentiation, and possibly an immutable characteristic.

In the next sections, we evaluate how the clockwork of hPSC might be unleashed and expanded in vivo; via the employment of alternate embryonic stem cell states with expanded embryonic potential, and differentiated over human-congruent developmental timelines within human–animal chimeras.

## The prospect for unlocking the full developmental potential of hESCs within optimized human–animal chimera systems

**Fig. 3:**
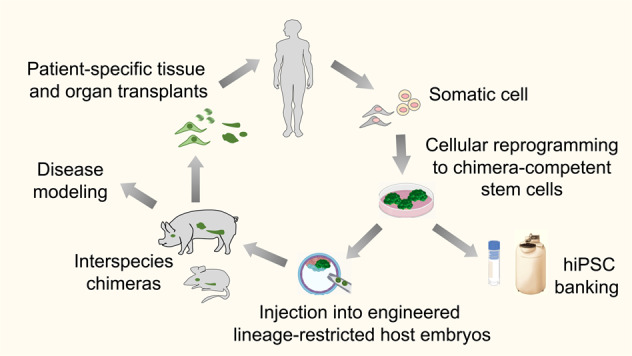
Interspecies chimera technology for producing patient-specific adult-staged tissues. Schematic of hiPSC-derived organogenesis via interspecies chimerism. Reversion of primed, conventional hPSCs into chimera-competent primitive epiblast-like or totipotent-like stem cell states may facilitate engraftment in pre-implantation staged animal embryos. Injection into genetically altered animal embryos using blastocyst complementation technologies may facilitate the generation of lineage-restricted human tissues or whole organs or for use in clinical transplantation.

Mouse ESCs produce developmentally complete and viable adult offspring following engraftment into either a blastocyst or morula-staged embryo host. This capacity for functional incorporation into a developing blastocyst is lost in inner cell mass (ICM)-derived cells following implantation^68^. Although injection of hPSC into human embryos cannot ethically be performed, the in vivo developmental capacity of hPSC, could in principle, be indirectly evaluated via the technology of blastocyst complementation (BC) within interspecies chimeras (Fig. 3). Moreover, adult tissues developed within such human–animal BC hiPSC chimeras could be leveraged to mitigate the shortage of transplantable patient-specific organs.

While this capacity has not yet been realized with hPSCs, whole organs have been derived with mouse-rat interspecies ESC systems^69–71^. For example, Kobayashi et al.^69^ employed BC to produce rat organs in the mouse species. Rat-PSCs (rPSCs) were injected into murine Pdx^−/−^ ICMs (which are incapable of developing a pancreas), and implanted into pseudopregnant mice uteri; live rat-murine chimeric pups were produced with a functional pancreas created entirely from rPSCs^69^. Unlike in vitro-directed differentiation, interspecific BC provided a developmental advantage to ESCs by providing them an empty niche to proceed to lineage-specific adult organogenesis. Another rat–mouse BC chimeras similarly generated interspecific hemato-vascular^70^ and kidney tissues^71^. Importantly, when an empty BC organogenesis niche was not provided, interspecific chimerism was highly inefficient and dependent on the species of both the injected donor ESCs and the host embryo recipient. For example, when rESCs were injected into wild-type (WT) mouse blastocysts (without BC), rat cells were found in early embryos, but their presence was extinguished following 11.5 days of gestation (E11.5)^72^. Although the etiology is unclear, the sudden disappearance of rat tissue in a developing host mouse embryo may relate to their differing developmental kinetics. Intriguingly, rESCs also failed to form chimeras with host pig embryos. Although unclear, it is possible that this result represented the opposite phenomenon: host porcine developmental kinetics were *too slow* for rapidly differentiating rESC^73^.

In contrast to post-implantation-derived murine EpiSC, only pre-implantation ICM-derived mESCs efficiently integrated with host blastocyst ICM cells to develop germline-competent chimeras^74^. These rodent chimera studies reinforced the notion that if human–animal BC strategies are to be realized, reversion of conventional, lineage-primed hPSC to a naïve epiblast-like state will be necessary. In addition, hPSCs and mEpiSCs both exhibit strong lineage-primed gene expression, interline differentiation variability, and compromised differentiation potency; all of which likely impede efficient embryonic chimerism. This may partially explain why conventional hPSC, which share molecular and functional similarity with primed murine EpiSC, displays limited capacity for chimerism with murine host blastocyst ICMs^75^.

Although standard-cultured mESC already possesses a high capacity for developmental contribution in morulae and blastocysts, chimera capacity can be improved with the LIF-2i naïve reversion method^76^. The LIF-2i method (i.e., LIF + MEK inhibitor + GSK3β inhibitor) established that heterogeneous mESC populations cultured in serum and LIF stabilized to a homogenous naive “ground state” via simultaneous blockade of MEK/ERK signaling and reinforcement of β-catenin signaling^77^. Although some primed mEpiSC lines retained naïve-like chimera contribution^78–80^, their poor baseline chimera inefficiency supports the notion that reversion to a naïve state is also necessary for primed hPSC for efficient human chimera-forming capacity.

The LIF-2i system alone was insufficient for reverting hPSC to a similar naïve pluripotent “ground state”^10^. However, several methods have now been reported which incorporated various modified versions of the classical LIF-2i method, but that supplement it with other small molecules; to sustain hPSC in a naïve mESC-like epiblast state (Table 1; Fig. 1)^10^. Nevertheless, despite using highly sensitive mitochondrial DNA PCR detection assays, these studies collectively revealed that injection of naïve-reverted hPSC into a mouse, rabbit, or ungulate blastocysts or morulae, or alternatively naïve-reverted non-human primate (NHP) rhesus PSC injected into a mouse, rabbit, or monkey embryos, resulted in limited human or monkey fetal chimerism (Table 1)^73,81–86^.

**Table 1:** Summary of human and non-human primate (NHP) interspecies chimera experiments.

Interspecies chimera experiment (reference) citation number	Donor cell origin	Host-species embryo	Genetic modification	Range of chimera efficiency	Interspecies chimera human lineage contribution				
					Embryo proper			Trophectoderm	Primitive endoderm
					Mesoderm	Endoderm	Ectoderm		
Gafni et al.^81^	N-hPSC	Mouse	–	–	Yes	N.A.	Yes	N.A.	N.A.
Theunissen et al.^84^	N-hPSC	Mouse	–	0.0005%	N.A.	N.A.	N.A.	N.A.	N.A.
Wu et al.^100^	rs-hPSC	Mouse	–	10–60 cells per embryo	Yes	Yes	Yes	N.A	N.A.
Wu et al.^73^	rs-hPSC	Pig	–	–	Yes	Yes	Yes	N.A	N.A.
Huang et al.^129^	P-hPSC	Mouse	*BMI* Overexpression	0.0001%	Yes	Yes	Yes	Yes	Yes
Hu et al.^91^	N-hPSC	Mouse	–	0.14–4.0%	Yes	Yes	Yes	N.A	No
Yang et al.^130^	hEPSC	Mouse	–	0.0001–0.01%	Yes	N.A.	Yes	Yes	N.A.
Das et al.^90^	P-hPSC	Pig	BCL Overexpression	0.0005%	Yes	N.A	N.A	No	No
Salazar-Roa et al.^131^	P-hPSC	Mouse	mir-203	1–3%	Yes	Yes	Yes	N.A.	N.A.
Wu et al.^100^	rs-NHP-PSC	Mouse	–	–	N.A.	N.A.	N.A.	N.A.	N.A.
Nowak-Imialek et al.^101^	NHP-PSC	Pig	–	–	N.A.	N.A.	N.A.	Yes	N.A.
Fu et al.^85^	Naïve NHP-PSC	Pig	–	0.001–0.0001%	Yes	Yes	Yes	No	No
Aksoy et al.^86^	Naïve hPSC, Naïve NHP-PSC	Mouse, Rabbit	–	undetectable	N.A.	N.A.	N.A.	N.A.	N.A.

*N-hPSC* naïve human PSC, *P-hPSC* primed human PSC, *rs-hPSC* region-specific human PSC, hEPSC human extended PSC, NHP-PSC nonhuman primate PSC.

**Fig. 4:**
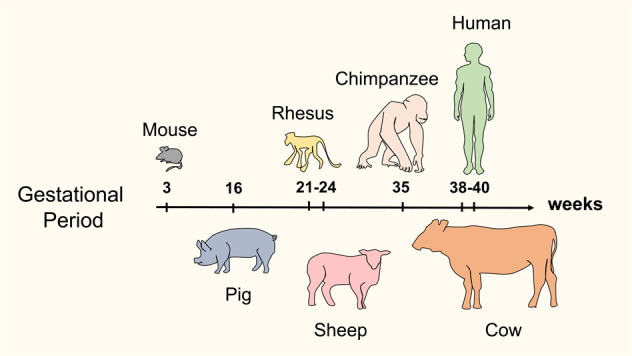
Comparative gestational periods of candidate hosts for human–animal interspecies chimera development. Mouse (18.5 days), pig (114 days), sheep (152 days), nonhuman primate (NHP) rhesus monkey (166 days), NHP chimpanzee (243 days), human (280), cow (283 days)^128^.

To test the hypotheses that longer, human-compatible gestational time-frames may be necessary for facilitating human–animal chimera development, larger mammals (e.g., pigs, sheep, and monkeys) were employed; along with an alternate, naïve-like hPSC states (Fig. 4)^87–89^ However, the problem of limited human tissue contribution to animal fetal development was still not improved. For example, following injection of N-hPSCs into pig blastocysts, efficiencies of only ~1 human cell per 10,000 animal host cells were reported, and only at up to 4 weeks of gestation^73,90^. Human cells were undetectable at later gestational time points. Hu et al.^91^ reported an improved N-hPSC culture system which promoted interspecific chimerism in more advanced murine embryos^91^. Nonetheless, despite the use of animal hosts with more human-congruent gestational developmental timelines, the overall chimeric contribution of human/NHP naïve PSC following injection into various pre-implantation animal embryos has thus far been minimal.

Interestingly, several interspecies chimerism studies have provided insight into the challenge of overcoming the innate disparate developmental kinetics between co-developing species. For example, an increase in tempo of neural lineage gene transcription was observed when hPSCs were cocultured with mPSCs. Although hPSC developmental kinetics of lineage gene transcription were incongruent with that of murine mPSC neural differentiation, there was a significant increase in their differentiation tempo when compared to control human hPSC differentiation^92^. This study provided the possibility that human developmental kinetics could be accelerated via in vitro culture manipulations between species with more rapid developmental kinetics. In contrast, in vivo chimeras between differing fish species followed independent species-specific development kinetics^93^.

## Human totipotent-like stem cells with expanded embryonic developmental potential may be necessary for unlocking efficient human–animal chimerism

The poor baseline functional pluripotency of current human naïve reversion methods may be one of the most significant obstacles currently impairing progress in human–animal chimerism technologies. Although the etiology of impaired functional pluripotency of current naïve reversion methods is unclear, it may derive from a reported chromosomal instability and aberrant erasure of genomic imprints^84,94–98^. In addition, inappropriate species-specific communication of developmental cues from cell–cell adhesion signaling barriers, and incongruence of innate developmental clocks (e.g., between mouse and human) may also impair efficient interspecific fetal development. For example, the divergence between human and murine embryogenesis may misdirect post-implantation morphogenesis, or produce antagonism in determining the appropriate ontogenetic size and shape of organs in the host animal.

Interestingly, Tachibana et al.^99^ identified an important species-specific factor that may uniquely impair successful embryonic chimerism in NHP (and perhaps also humans): a more primitive, pre-naïve epiblast developmental stage of the injected embryonic stem cell may be necessary for efficient chimerism. In their study, NHP rhesus chimeras were created by injecting either unmanipulated ICM or cultured (primed) rhesus PSC into NHP rhesus blastocyst hosts of the *same species* (intra-specific chimeras). Surprisingly, unlike rodents, injection of NHP-ICM cells (composed presumptively of unmanipulated, pristine, naïve rhesus PSC) into intraspecific NHP hosts did *not* produce full chimeric NHP embryos. Indeed, rhesus ICMs contributed only to extraembryonic tissues, as well as to the spleen and fetal liver in limited amounts of developing rhesus monkeys. In contrast, successful NHP-chimeric rhesus embryos and mature animals have generated *only* with the aggregation of developmentally more primitive 4-cell (totipotent) rhesus blastomeres. These results suggested that naive epiblast-like NHP-PSC possess inherently less embryo chimerism potential than do rodent ICM cells, suggesting the hypothesis that alternative, totipotent-like stem cells may be required for efficient NHP (or human) chimerism. These data, at least in part, may account for why naïve hPSCs have met limited success in generating human interspecific organogenesis within animal embryos^73,74,81^. At a minimum, the limited proficiency for generating chimeras with primed rhesus PSC in these and other studies aligned with the principle that primed EpiSC are incompetent in chimera formation in pre-implantation murine embryos^86,100,101^.

In any case, the genomic imprinting aberrations of various human naïve reversion systems may alone account for their poor functional pluripotency. These aberrations resembled those reported in LIF-2i-reverted mESC that resulted in poor differentiation and chimera development^102^. Eroded imprints in naïve mESC were attributed to *prolonged* exposure to the MEK inhibitor PD0325901; which is a critical component in the LIF-2i cocktail for stabilizing the undifferentiated naïve state. Interestingly, human naïve culture systems that incorporated prolonged MEK inhibition in their naïve reversion cocktails also displayed high frequencies of abnormal karyotypes, a systematic erasure of parental genomic imprints, and repressed directed differentiation capacities^84,94–98^. For example, 5i/L/A-reverted N-hPSC required prolonged culture from naïve culture conditions back to primed conditions to re-acquire and rescue differentiation competency^84,94^. Such impaired functional pluripotency of human naïve states is in contradiction with murine PSC data^103–107^. For example, although primed mEpiSC displayed highly variable differentiation capacity and lineage-primed gene expressions^103,104,108^, naïve mESC exhibited augmented neuroectodermal differentiation potency that generated neural cells that more closely resembled those from the mouse adult brain^103–105^.

**Fig. 5:**
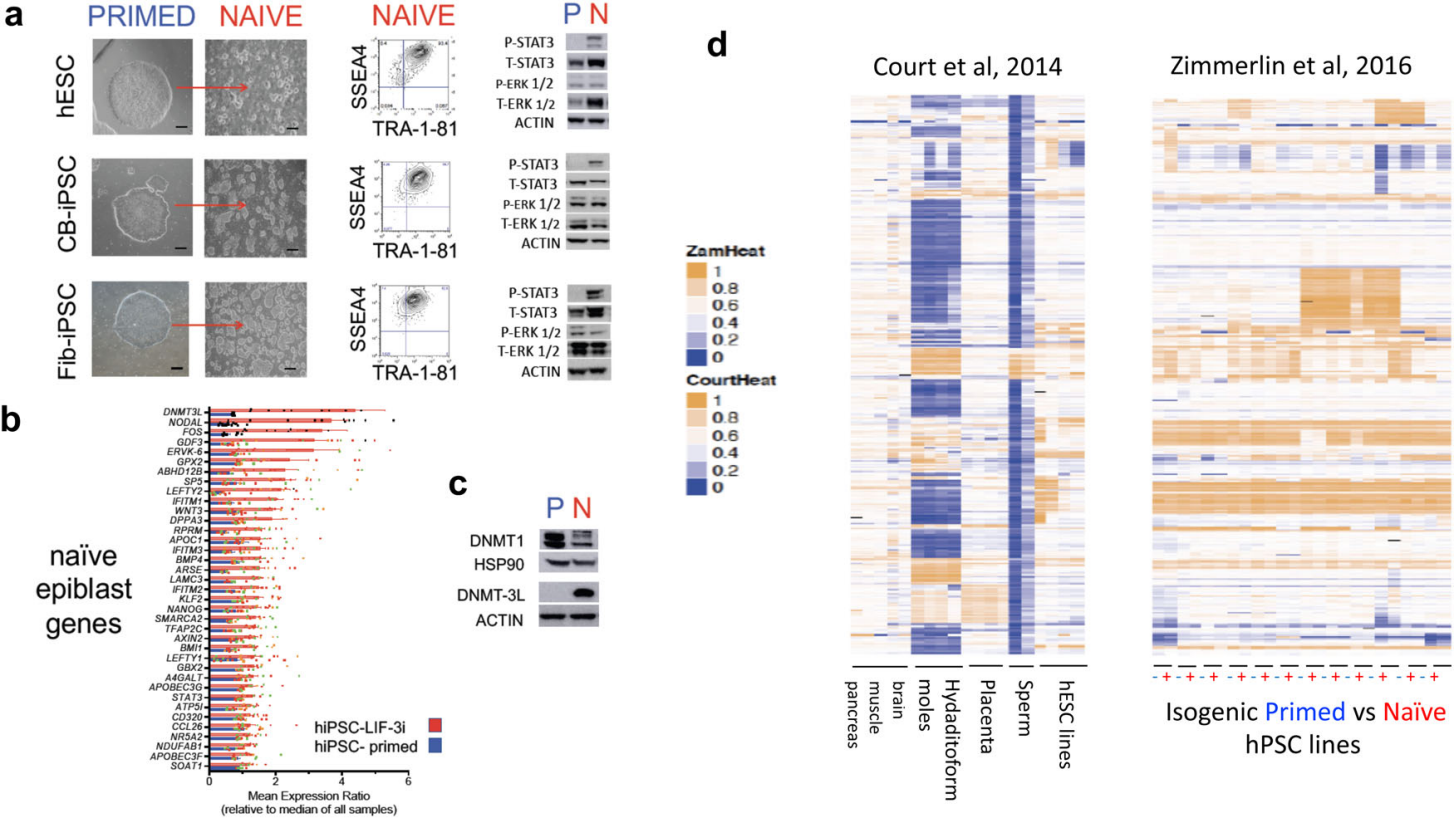
Chemical reversion of conventional primed hPSC with a tankyrase/PARP inhibitor promotes stable rewiring to a human preimplantation epiblast-like state with intact epigenomic imprints. The TIRN method is a two-step culture system^109,111^ comprised of one brief adaptation step of conventional hPSC with LIF and five small molecules (LIF-5i) that includes XAV939 (tankyrase/PARP inhibition), CHiR99021 (GSK3β inhibition), PD0325901 (MEK inhibition), forskolin (adenylate cyclase activation), and purmorphamine (Hedgehog signaling activation); followed by continuous, stable culture in *only* LIF/XAV939/CHIR99021/PD0325901 (LIF-3i) for at least 30 passages. This tankyrase/PARP inhibitor-mediated modification of the classical LIF-2i method has been validated to stably revert over 30 hPSC lines, and is independent of genetic donor background^109,111^. **a** (Left panels) Three representative hPSC lines^109,110^ (RUES1 hESC, cord blood (CB)-hiPSC 6.2, and fibroblast (fib)-hiPSC C.2 are shown in their starting primed (i.e., E8 medium; PRIMED) conditions, and 6–10 passages post culture in continuous TIRN-reverted (NAÏVE) conditions^109,111^. Monolayer bFGF-dependent hPSC colonies in primed conditions became tolerant to bulk single-cell passaging and acquired a typical dome-shape morphology following TIRN reversion. (Middle panels) TIRN-hPSC retained strong expression of TRA-1–81 and SSEA4 surface antigens by flow cytometry. (Right panels) Western blot analyses in these three primed (P) and TIRN (N) lines demonstrated that TIRN-hPSC acquired de novo expression of phosphorylated STAT3 and reduced ERK1/2 phosphorylation. **b** TIRN-hPSC uniformly shifted their transcriptomes toward a pre-implantation naïve epiblast identity. Mean expression ratios of naïve epiblast-specific genes from Illumina gene arrays from isogenic primed-to-naïve-reverted pairs of genetically independent hPSC (*n* = 9 lines)^109^. **c** Western blot analysis of primed (P) and TIRN (N) CB-hiPSC (E5C3). TIRN-CB-hiPSC markedly upregulated naïve epiblast-specific DNMT3L whilst *maintaining* DNMT1 protein expressions following TIRN reversion^109,110^. **d** Infinium CpG methylation heatmaps of genomic Imprinted promoter regions of a panel of primed and TIRN-reverted isogenic hPSC samples revealed that TIRN-hPSC retains normal somatic epigenomic imprint configurations, similar to their isogenic primed states. Heatmaps compare previously published Zambidis lab^109^ methylation data alongside published Court et al.^121^ data of identical imprinted genomic regulatory regions as controls. Zambidis lab methylation beta values were subset to exact imprinted regions provided by Court et al.^121^ (methylation beta values: 0—completely hypomethylated probe; 1—completely methylated probe). Matching Infinium probes are sorted by chromosomal location and arranged into their adjacent primed (−) and naïve (+) hPSC isogenic pairs. The heatmaps of the same imprinted regions from Court et al.^121^ include abnormal androgenetic hydatidiform mole, normal human tissues (e.g., brain, muscle, placenta, and sperm), and primed hESC lines.

In contrast, Zimmerlin et al.^109^ described an alternative small molecule-based method that stably reverted conventional primed hPSC to a human naïve epiblast-like state *without* compromised differentiation potency and without epigenomic aberrations at imprinted regions^109^. This method supplemented the classical LIF-2i cocktail^77^ with the pleiotropic tankyrase/PARP (poly-ADP-ribose polymerase) inhibitor XAV939 (LIF-3i) (Fig. 5). This chemical tankyrase/PARP inhibitor-based method rapidly and stably reverted a broad repertoire of genetically independent conventional, lineage-primed hESC and hiPSC lines to adopt transcriptional, epigenetic, and biochemical features of the human pre-implantation epiblast (Fig. 5a, b). Tankyrase/PARP inhibitor regulated naïve hPSC (TIRN-hPSC) possessed multiple naïve epiblast-like ICM characteristics that included MEK-ERK/bFGF signaling independence, activated phosphorylated JAK/STAT3 signaling, distal OCT4 enhancer usage, global DNA CpG hypomethylation, and increased expression of activated beta-catenin^109,110^. Importantly, in contrast to other naïve reversion methods^84,94–97^, Zimmerlin et al.^109^ reported that supplementation of the core LIF-2i cocktail with *only* this tankyrase**/**PARP inhibitor (XAV939) protected a wide repertoire of genetically independent TIRN-hPSC lines against the erosion of CpG methylated genomic imprinted regions. In addition, we show here that TIRN-hPSC also maintained their DNMT1 expression (Fig. 5c, d). Moreover, TIRN-hPSC possessed reduced lineage-primed gene expression and did not require reversion culture back to primed culture conditions prior to differentiation^109–111^. The human TIRN system is the only human naïve reversion method described thus far that generates naïve epiblast-like hPSC with expanded multi-lineage differentiation potency, normal epigenomic imprints, and improved in vivo survival of differentiated progenitors^109–112^. TIRN-hPSC was recently shown to generate fully differentiated vascular progenitors with improved functional and long-term engraftment capacities in vivo that were superior vascular progenitors generated by normal or diseased primed, conventional hiPSC^110^.

The performance of the TIRN system in interspecies chimeras has not yet been reported. However, Yang et al.^113^ reported a chemical cocktail analogous to Zimmerlin et al’s original core LIF-3i system, that similarly supplemented the base LIF-2i cocktail with XAV939, which has non-specific and pleiotropic PARP inhibition activities. Their small molecule cocktail was used to potentiate the self-renewal of murine blastomeres and reverted them into “expanded potential stem cells” (EPSC) with significantly improved murine chimerism potential following blastocyst injection. These murine EPSC contributed to both embryonic and extra-embryonic lineages (trophectoderm and primitive endoderm) in embryonic chimera experiments^113–115^. Interestingly, several human naïve PSC studies have also employed the sole use of the multi-PARP inhibitor XAV939 (as a WNT pathway inhibitor) to rescue the differentiation block of other human naïve states generated with small-molecule cocktails that incorporated prolonged MEK inhibition^116,117^.

Collectively, these studies promote the concept, originally proposed by Zimmerlin et al., that tankyrase/PARP enzymatic inhibition likely promotes global cellular processes in stem cells that expand their functional pluripotency and protect against epigenomic instability^112^. For example, tankyrase/PARP inhibitor-based culture promoted erasure of dysfunctional epigenetic donor cell memory, and possibly disease-associated aberrations in patient-specific TIRN-hiPSC^110^. Thus, TIRN-hPSC may represent a new class of stem cells with improved epigenetic plasticity and multi-lineage differentiation potential, that may have high impact for both regenerative therapies and the generation of human–animal chimeras^110,112^.

## Conclusions and ethical/regulatory considerations

We have summarized the potential obstacles that limit the generation of adult human tissues from hiPSC using in vitro differentiation paradigms. We proposed a ‘developmental incongruence’ hypothesis that states that differentiation of conventional hPSC generates embryonic stage cells, and that derivation of clinically relevant adult cells and organs is ultimately limited with in vitro approaches alone (including via three-dimensional organoid strategies). We argued that niche signals provided during long-term normal fetal and adult organogenesis are too complex to mimic within short-term ex vivo cultures. We posited that directed ex vivo differentiation of hPSCs follow cell-autonomous, innate species-specific developmental clocks, and in vitro manipulations of signaling pathways alone are unable to accelerate them. We advocate that interspecies chimerism is a solution to these obstacles, and if optimized, could unlock the full potential of hiPSC by facilitating the developmental timelines required for adult cell fate decisions.

Thus far, NHP–NHP, NHP–animal, and human–animal interspecific strategies have yet to match the success of rat-mouse studies^69,73,118^. If successful, hiPSC-based human–animal interspecific chimera technologies could provide transplantable patient-specific adult tissues or whole human organs, as well as the creation of more accurate humanized animal models of disease. However, several barriers currently interfere with the efficient interspecies chimeric contribution of hPSC and result in poor integration, survival, and expansion of hPSC in embryonic animal hosts^119^. These barriers include inappropriate developmental matching between donor hPSC and the recipient animal embryo, ineffective functional naïve pluripotency due to chemical reprogramming-associated epigenetic aberrations, inefficient cell–cell interactions between co-developing human and animal organs, and potentially multiple other unknown obstacles. The quality and functional performance of alternative stem cell states, and a stringent observance of species-specific differentiation kinetics may ultimately overcome these obstacles. However, important challenges remain in deriving more primitive human and non-human stem cells. For example, stable ground state naïve PSC have not been easily captured in culture in all species. Furthermore, the capture of bonafide, self-renewing totipotent stem cells appears inherently meta-stable in culture and has not yet been reported for any species.

Future efforts should focus on improving allogeneic NHP chimera systems (e.g., monkey-to-monkey)^83^, and large animal (i.e., pig–pig) allogeneic intraspecific chimeras^89^. Such efforts may also enlighten novel approaches for allogeneic tolerance induction to porcine organs to advance the parallel field of xenotransplantation. We also proposed that efficient human–animal chimerism may ultimately require the derivation of human totipotent-like stem cells with expanded embryonic/extra-embryonic potency. In addition, these goals will benefit from novel BC strategies (Fig. 3) that facilitate human tissue development within engineered lineage-restricted animal embryo hosts with human-compatible gestational time-frames (Fig. 4).

Overall, we have reinforced the concept that immutable and species-specific developmental clocks ingrained within human stem cells may ultimately determine the functional incorporation of human cells within recipient hosts. Further basic experimental work should investigate how immutable factors that regulate adult developmental clocks (e.g., protein stability and cell cycle) can be manipulated; perhaps via genetic alteration of developmental tempo-determining transcriptional factors. A new frontier of stem cell-based tissue engineering may lie in the manipulation of these innate developmental clocks for producing patient-specific adult cells and whole organ replacement. A framework to explore these questions should consider the species-specific tempo of differentiation in not only the chronological sense of hours, days, weeks, or months, but rather the maintenance, codification, and even unlearning of in vivo environmental cues. By conceptualizing developmental tempo in this fashion, it may be possible to elucidate the correlative nature of cell identity and time and allow for the possibility of accelerating, or even reversing adult human developmental kinetics, possibly within interspecies chimera platforms. If successful, optimized interspecific chimeras that master these immutable developmental clocks may ultimately eliminate the variability inherent within in vitro differentiation protocols.

In addition to the scientific challenges outlined above, there currently remain many regulatory limitations that prohibit meaningful progress of human–animal chimera technologies in the US; including a 2015 moratorium on NIH funding for such research. Moreover, although strict restrictions for introducing hESC into NHP or human embryos are already defined by the National Academy of Sciences (NAS), the International Society of Stem Cell Research (ISSCR) has condoned some categories of human–animal embryonic chimera research, prohibited other types, and placed all of them under the purview of institutional scientific and ethical review boards. For example, many jurisdictions specifically prohibit the introduction of hPSC into NHP embryos, or into animal systems that result in germline contribution of human gametes or “higher brain function”^120^. Research on genetically modified animal models that address and abate these ethical concerns should be encouraged, with appropriate assurances and humane safeguards of animal welfare. Finally, interspecific chimera research may provide an alternative source of human fetal tissues for biomedical research (i.e., harvested from chimeric human–animal feti). Such tissues are currently inaccessible due to federal research funding restrictions, and yet are critical for the conduct of important work in understanding human developmental disorders.

In conclusion, we advocate for the establishment of national and international oversight guidelines that enable this important area of biomedical research to proceed in an ethical manner, thus creating an environment for lifting its current federal funding restrictions. The creation of a scientific and regulatory framework for performing interspecies chimera research can positively and humanely advance the important goals of regenerative medicine.

## Methods

### Conventional primed and TIRN cultures of hESC and hiPSC lines

The hESC line RUES01 was obtained from the Wisconsin International Stem Cell Bank (WISCB). All hESC experiments conformed to guidelines outlined by the National Academy of Sciences, and the International Society of Stem Cell Research (ISSCR). This commercially acquired hESC line was under the purview of the Johns Hopkins University (JHU) Institutional Stem Cell Research Oversight (ISCRO), and conformed to Institutional standards regarding informed consent and provenance evaluation. CB-derived hiPSC (CB-hiPSC) lines 6.2 and E5C3, and fibroblast-derived hiPSC (fib-hiPSC) line C.2 were all previously described^109,110^. All hESC and hiPSC lines were maintained in undifferentiated conventional, feeder-free primed states in Essential 8 (E8) medium and naive-reverted with the LIF-5i/LIF-3i TIRN system, as described^109–111^.

### Flow cytometry

Pluripotency surface marker analysis of hPSC was performed as previously described^109–111^. hPSC single-cell suspensions were filtered through a 40 µm cell strainer, centrifuged (200 g; 5 min), resuspended in culture medium, and washed. 100,000 cell aliquots were incubated for 20 min on ice with either isotype controls or monoclonal mouse antihuman antibodies SSEA1-APC (5 µL, 551376, BD Biosciences), SSEA4-APC (5 µL, FAB1435A, R&D Systems), TRA-1-60-PE (10 µL, 560193, BD Biosciences) and TRA-1-81-PE (10 µL, 560161, BD Biosciences). Samples were fixed overnight in PBS, 0.25% Formaldehyde (Affymetrix) prior to analysis. Fluorescence detection (at least 10,000 events per sample at an acquisition rate below 300 events per second) was performed with a FACSCalibur cytometer (BD Biosciences) equipped with blue argon (488 nm) and red diode (635 nm) lasers and BD CellQuest Pro analytical software. Analysis was performed using FlowJo software (Tree Star).

## Western blots

All blots derived from the same experiment were processed in parallel. Antibodies and blotting methods were previously described^109–111^. Cells were collected from either isogenic primed (E8 media on vitronectin-coated plates) or TIRN-reverted (LIF-3i/MEF plates) conditions using either Versene (ThermoFisher, 15040-066) or Accutase (Gibco A1110501). MEF was removal from TIRN cultures by pre-plating on 0.1% gelatin-coated plates for 1 h. Cells were washed once in PBS, pelleted with gentle centrifugation, snap-frozen in liquid nitrogen, and then lysed in 1× radioimmunoprecipitation assay buffer (ThermoFisher Scientific, 89900) supplemented with 1.5 mM EDTA and 1× Protease Inhibitor (ThermoFisher Scientific, 78430). Cell lysates were quantified using the Pierce BCA assay method (ThermoFisher Scientific). 25μg of protein cell lysate per sample was equally loaded on a 4–12% NuPage Gel (ThermoFisher Scientific, NP0336). The gel was transferred using the iBlot2 system (Life Technologies), and nitrocellulose stacks (Invitrogen). Membranes were blocked in Tris-buffered saline (TBS), 5% non-fat dry milk (Labscientific), 0.1% Tween-20 (TBS-T) for 1 h. Blots were incubated overnight at 4 °C with the following primary antibodies according to manufacturer’s instructions: anti-STAT3 (BD Biosciences, clone 84/Stat3, Cat# 610189; 1:1000), anti-phospho-STAT3 (Cell Signaling, clone 3E2, Cat# 9138; 1:1000), anti-p44/42 MAPK (Erk1/2) antibody (Cell Signaling, Cat#4695; 1:1000), anti-phospho-p44/42 MAPK (Erk1/2); Thr202/Tyr204 (Cell Signaling; Cat#4370, 1:2000), anti-DNMT1 (Cell Signaling, Cat#5032, 1:1000), anti-DNMT3L (Abcam, Cat#ab194094, 1:1000), anti-beta-Actin (Abcam, clone AC-15, cat# Ab6276; 1:5000), anti-HSP-90 (Cell Signaling, Cat#4877, 1:1000). Anti-actin or anti-HSP90 antibody staining was performed on each membrane, as a loading control. Membranes were rinsed three times in TBS-T, incubated with horseradish peroxidase-linked goat secondary antibodies (Cell Signaling) for 1 h at room temperature, rinsed three times, and developed using Pierce ECL Substrate (ThermoFisher Scientific, 32106). Chemiluminescence detection was imaged using an Imager 600 (Amersham).

### Bioinformatics gene expression and genomic imprinting methylation array analyses

The NIH accession numbers of expression data analyzed in this manuscript were previously published: GSE44430^109^. Methylation beta values of processed Illumina 450k methylation arrays were obtained from GEO archived matrix sheets from Zimmerlin et al. GSE65214^109^, and Court et al. GSE52578^121^. Overlapping regions between the Illumina 450k methylation array and the imprinted regions provided from the Court study were found using the Bioconductor R GenomicRanges package and IlluminaHumanMethylation450k.db. Heatmaps were generated using the Bioconductor R ComplexHeatmap package.

## Supplementary information

raw western blots

## Data Availability

All relevant primary data is available from the authors. Raw Western blot source images for Fig. 5 are available online as Supplemental Information. The bioinformatics source data analyzed in this manuscript were from previously published experiments, and are available through the following NIH accession numbers: GSE44430^109^, GSE65214^109^, and GSE52578^121^.
