# Supplementary material for: Running the full human developmental clock in interspecies chimeras using alternative human stem cells with expanded embryonic potential
Source: NPJ Regen Med. 2021 May 17;6:25. doi: 10.1038/s41536-021-00135-1 (PMC8128894; doi:10.1038/s41536-021-00135-1)

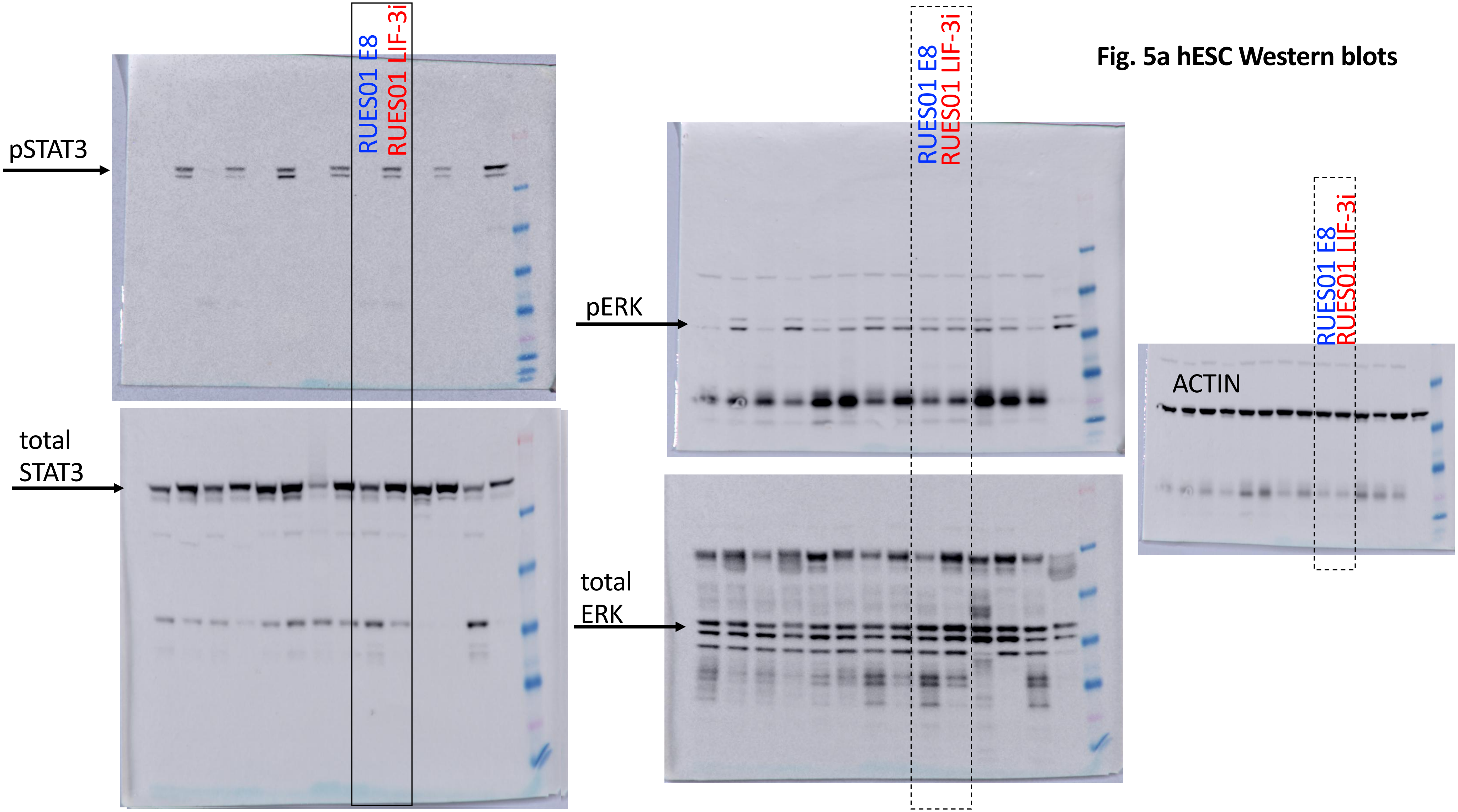

Fig. 5a hESC Western blots

Fig. 5a
CB-iPSC Western blots

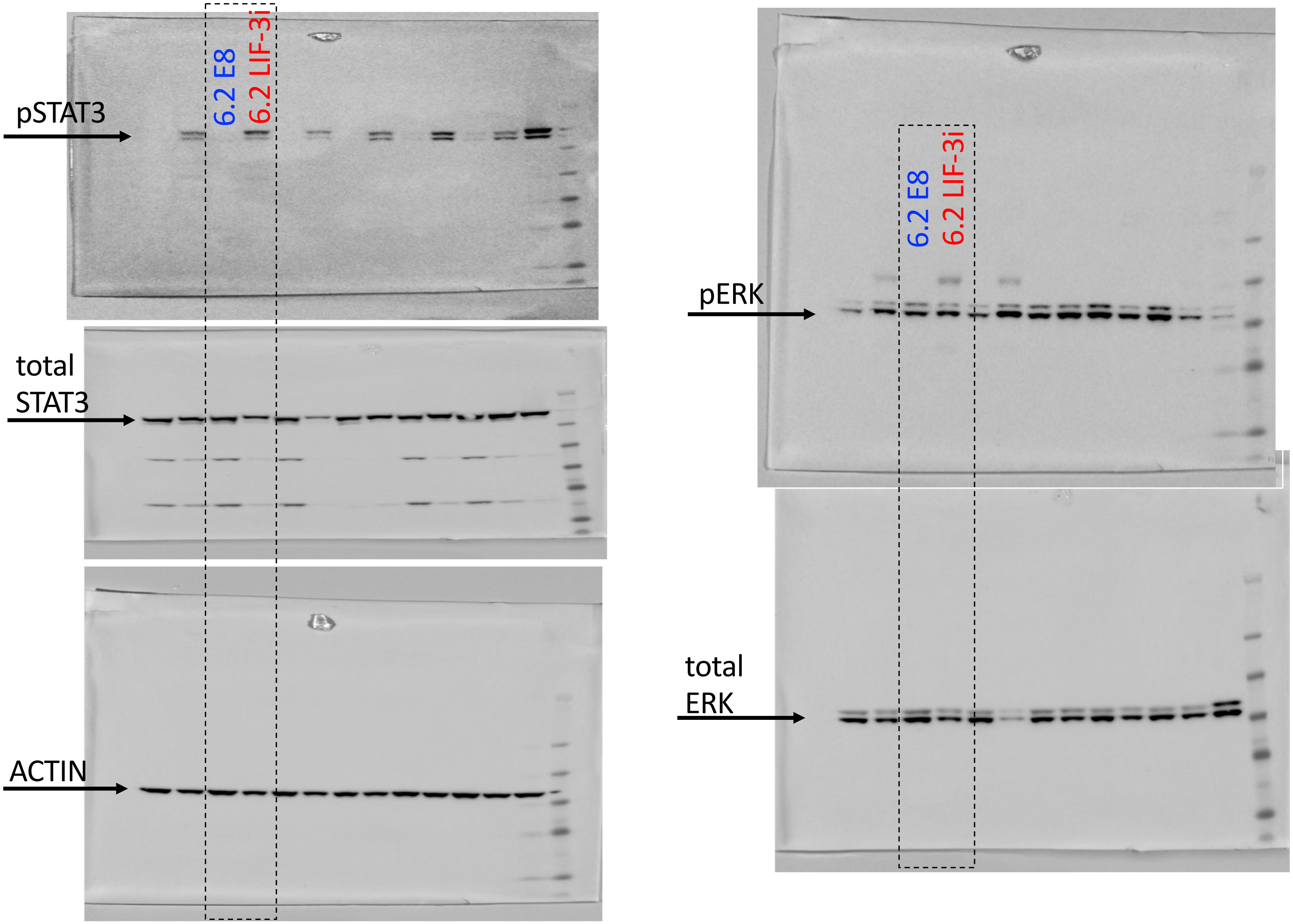

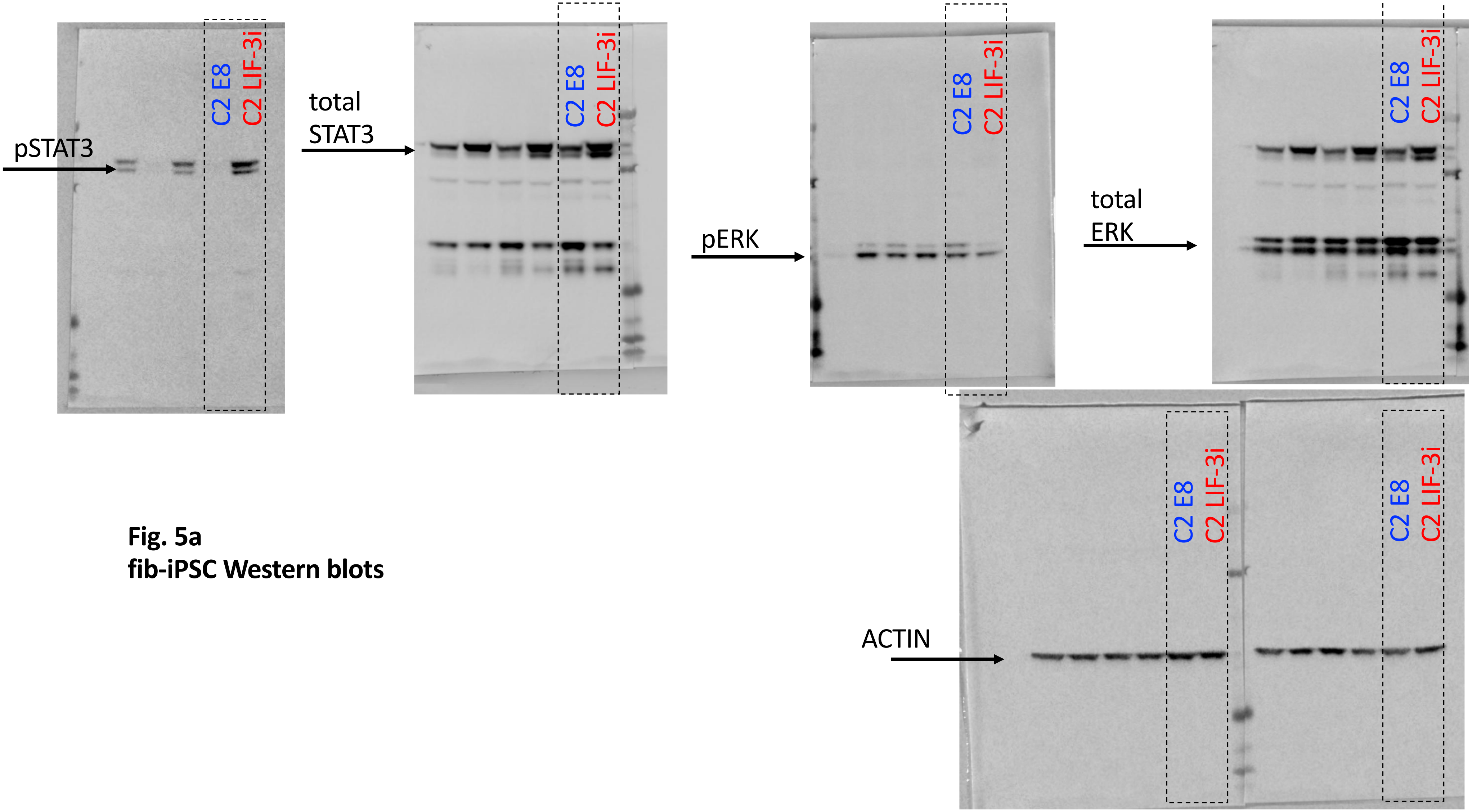

Fig. 5a
fib-iPSC Western blots

Fig. 5c
Western blots

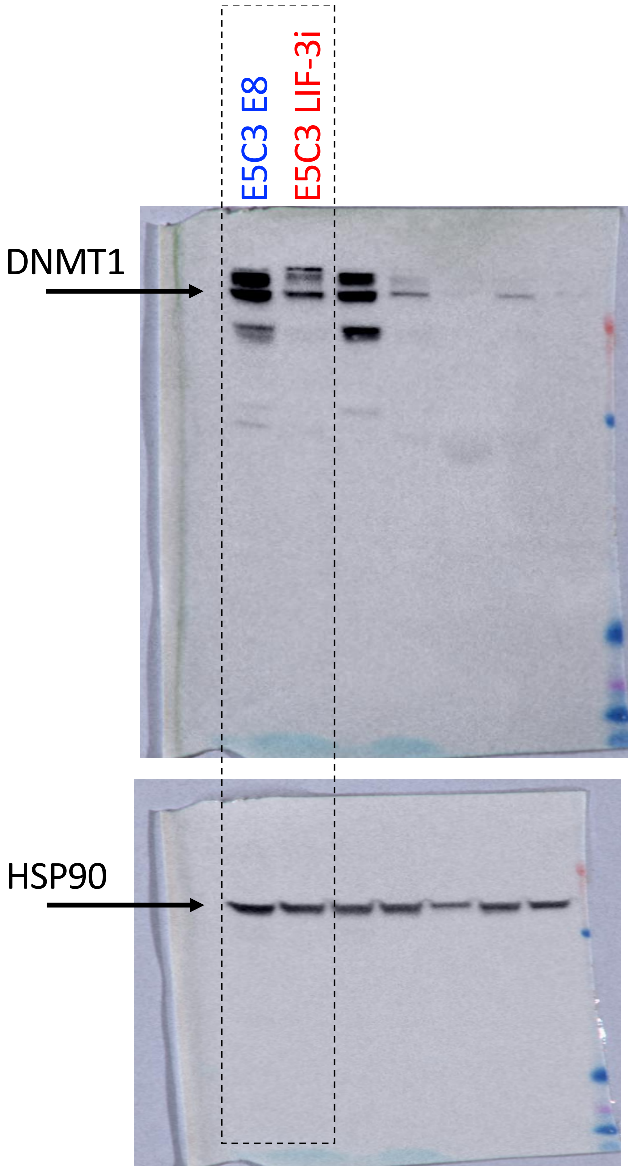

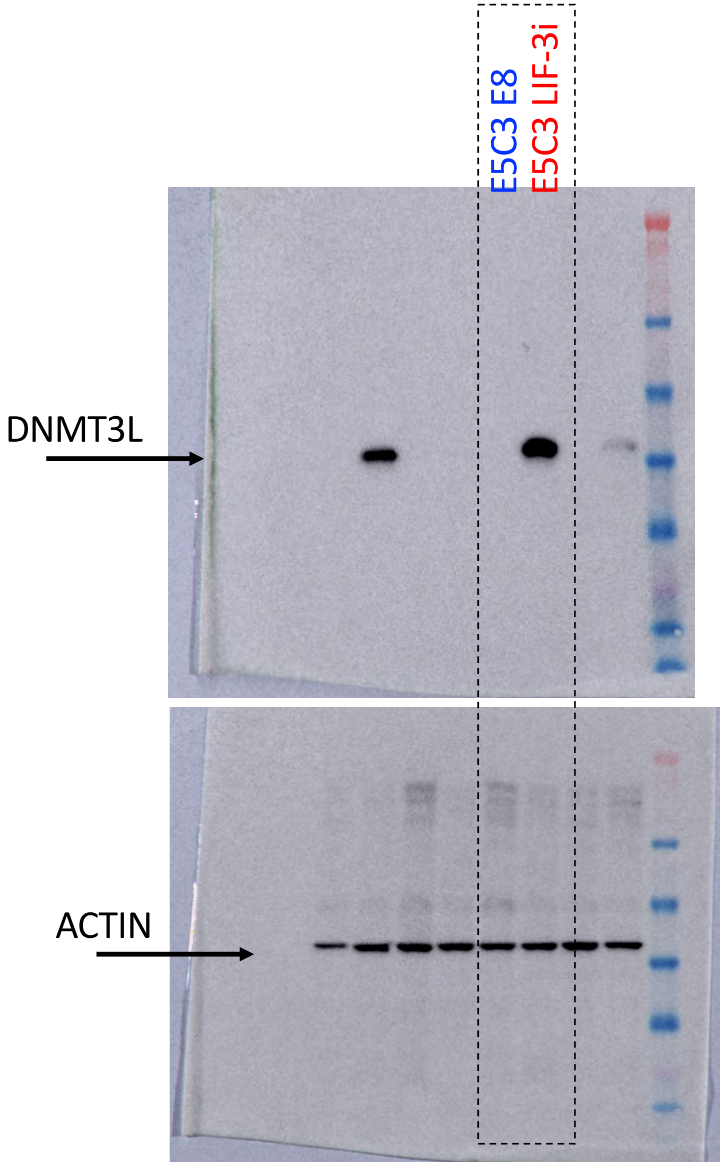

Supplement: Supplementary file 1 — raw western blots [file 41536_2021_135_MOESM1_ESM.pdf]
